# Vulnerabilities in snakebites in Sao Paulo, Brazil

**DOI:** 10.1590/S0034-8910.2015049005839

**Published:** 2015-11-10

**Authors:** Maria Rita Bertolozzi, Camila Morato da Conceição Scatena, Francisco Oscar de Siqueira França

**Affiliations:** I Departamento de Enfermagem em Saúde Coletiva. Escola de Enfermagem. Universidade de São Paulo. São Paulo, SP, Brasil; II Pós-Graduação em Ciências da Saúde. Faculdade de Medicina. Universidade de São Paulo. São Paulo, SP, Brasil; III Departamento de Moléstias Infecciosas e Parasitárias. Faculdade de Medicina. Universidade de São Paulo. São Paulo, SP, Brasil

**Keywords:** Snake Bites, Bothrops, Vulnerable Populations, Health Vulnerability, Socioeconomic Factors, Qualitative Research

## Abstract

**OBJECTIVE:**

To describe elements of vulnerability of victims of snakebite.

**METHODS:**

This qualitative, descriptive, cross-sectional study had, as theoretical framework, the concept of vulnerability in individual, social, and programmatic dimensions. We interviewed 21 patients admitted into a hospital specialized in the care of accidents caused by venomous animals. The interviews were analyzed according to a discourse analysis technique.

**RESULTS:**

Patients were mainly young men, living in remote countryside areas, where health services frequently have limited resources. We found social and individual conditions of vulnerability, such as precarious schooling, low professional qualification, housing without access to piped water, no sewage treated, and no regular garbage collection, and lack of knowledge on this health problem. Regarding the programmatic dimension, we found limited accessibility to the health services that could affect the prognosis and the frequency of sequelae and deaths.

**CONCLUSIONS:**

Considering such vulnerabilities evoke the need to improve the program for control the Accidents by Venomous Animals and the training of health workers, we highlight the potential use of the concept of vulnerability, which may amplify the understanding and the recommendations for the practice and education related to snakebites.

## RESUMO

**OBJETIVO:**

Descrever elementos de vulnerabilidade de vítimas de acidentes por serpentes.

**MÉTODOS:**

Este estudo qualitativo, descritivo e transversal teve como referencial teórico o conceito de vulnerabilidade, nas dimensões: individual, social e programática. Foram entrevistados 21 pacientes internados em hospital especializado no cuidado a vítimas de acidentes por animais peçonhentos. Os depoimentos foram analisados segundo técnica de análise de discurso.

**RESULTADOS:**

Os pacientes eram, em sua maioria, homens jovens e residentes em área rural, onde frequentemente os recursos de saúde são limitados. Foram observadas condições de vulnerabilidade individual e social, tais como: escolaridade precária, baixa qualificação profissional, moradia sem acesso à água encanada, esgoto e coleta regular de lixo e precário conhecimento a respeito desse agravo à saúde. Quanto à dimensão programática, verificou-se acessibilidade restrita ao serviço de saúde, que poderia repercutir no prognóstico e na frequência de sequelas e mortes.

**CONCLUSÕES:**

A constatação de elementos de vulnerabilidade em suas três dimensões evoca a necessidade de melhoria de programa de controle e formação dos trabalhadores de saúde. Destaca-se a potencialidade do uso do conceito de vulnerabilidade, o que pode ampliar a interpretação e recomendações para a prática e ações educativas relacionadas aos acidentes ofídicos.

## INTRODUCTION

Snakebites cause significant mortality rates in most tropical countries. In 2009, the World Health Organization (WHO) included snakebites in the group of neglected diseases.^29^ ^,^ ^30^ This health problem affects mainly people who live or work in rural areas, where generally health services are insufficient, with limited capacity of resolution and obtaining antivenom.^15^ Deficient and inadequate training of health professionals to identify snakes, recognize the signs and symptoms that lead to the diagnosis, and classify the poisoning and the appropriate treatment are features that contribute to the size of the problem.^15^

About five million accidents involve snakes yearly, resulting in 2.5 million poisonings and 125,000 deaths, also considering the amount of patients with permanent sequelae may be at least three times the amount of deaths.^8^ In Brazil, snakebites began to be regularly reported to the Ministry of Health by 1986. Currently, about 28,000 accidents caused by poisonous snakes are notified every year, 90.0% of them being caused by snakes of the *Bothrops* genus and approximately 9.0% by the genus Crotalus. In 2013, 78 of the 116 deaths by snakebites were caused by snakes belonging to the genus Crotalus.^a^ The most common species, and the main cause of accidents in the Southeastern Brazil, is the *Bothrops jararaca*. This species is agile, rises easily in bushes and low roofs, has high adaptive capacity, thrives in urban and wild environments, and present crepuscular and nocturnal activities.^13^ ^,^ ^21^ ^,^ ^24^ ^,^ ^26^ All patients with clinical manifestations or laboratory abnormalities, or both, because of snakebites ought to receive the antivenom as soon as possible, but many of them receive the antivenom too late. Generally, patients who suffer from this accidents present vulnerabilities that have not been investigated by previous studies, especially when the empirical material come from the patient’s point of view.

This study aims to evaluate vulnerabilities in snakebites to improve health care and also to disseminate preventive actions. Thus, the research questions if victims of snakebites have vulnerabilities.

The term “vulnerability” has been used in recent years, especially after the 1980s, in several epidemiological studies.^20^ The importance of studying bothropics accidents interpreted by the concept of vulnerability allows us to integrate dimensions concerning individuals, health policies, and social contexts. Ayres et al^2^ (2007) structured the concept of vulnerability, proposing that it is integrated by three dimensions. The first dimension is the individual, referring to the degree and quality of information and knowledge that people have on health problems, and their application in practice; the second dimension is the social, including the evaluation of the information obtained, the access to different kinds of communication, the availability of cognitive and material resources, and the power individuals must hold to participate in political and institutional decisions. The third one is the programmatic, concerns the evaluation of programs of disease control and incorporates the degree and quality regarding institutions’ commitment, management, resources, and the monitoring of programs in different levels of health care. The model of vulnerability not only accepts the traditional biological model, but also recognizes and strives to exceed it.^20^

The objective of this study was to describe the elements of vulnerability of snakebite victims.

## METHODS

The object of the study was delimited in the vulnerability theoretical framework.^1^ This option was based on the understanding that snakebites are not limited to individual exposure to the animal, but stems from a series of situations and contexts involving life, work, accessibility to health services, among others that will determine the occurrence, evolution, and accident severity. This is a hermeneutic, qualitative, descriptive, and cross-sectional study.

The study population was chosen among victims of bothropic accidents admitted into a hospital specialized in the care of accidents caused by venomous animals in Sao Paulo, SP, Southeastern Brazil. All victims were patients of above 15 years of age who spent more than 24h in the hospital and that presented mild or moderate classification at the admission, according to the criteria of the Brazilian Ministry of Health.^13^

Patients who presented cognitive deficit or accident classified as severe were excluded from participating.^13^

The data were collected from June 2010 to February 2012 since accidents of this kind are more common during spring and summer. Twenty-one subjects were integrated into the study and the definition of the sample was made by the recurrence of facts reported in the interviews. Patients were invited to be interviewed 24h after their admission.

The instrument used in the interviews was composed of two parts: the first was structured in closed questions, containing elements of social and demographic characteristics. The second was semi-structured, which allowed the patient’s free speech to seek the understanding of the actions taken in the moment of the accident, and how they perceived the accident, among other aspects that integrate the concept of vulnerability. The questions of this second part were: “Tell me how the accident occurred?”; “What was done at the site of the bite?”; “Is it possible to avoid being bitten?”; and “How was the assistance in the health unit?”.

This was an in depth semi-structured interview to explore the patients’ experiences and how they were understood by them.^28^ All interviews were recorded on the infirmaries of the hospital by one of the authors of the present article, who never had any previous relationship with the patients, ensuring free speech. It is worth to highlight that such places were always looked to maintain the patients’ privacy.

Sociodemographic data were systematized in Excel. Data related to the semi-structured part of the study were transcribed fully, and analyzed according to the technique of discourse analysis proposed by Fiorin and Savioli^3^ ^,^ ^9^ ^-^ ^11^ and adapted by Car^6^ by basing on the *Theory of Greimás: the Construction of Meaning Making*, whose themes and figures are extracted from testimonials. Systematic readings of the statements were done and then concepts and thematic categories were identified.

The analysis was performed by one of the researchers who have experience in this kind of qualitative research.^20^ The validation of the analysis was done by the interviewer and the discussion of the results was undertaken together with the other authors. We attended to the qualitative research review guidelines – RATS.

The study was approved by the Committee of Research Ethics of the Universidade de São Paulo (Process 319/10, in April 13, 2011). All interviewed were informed orally and in writing about the objectives of the study, and signed consent forms. All precautions related to the confidentiality of the interviewed were adopted.

## RESULTS

Patients were mostly young men, aged between 21-50 years (57.1%), with 6-10 years of schooling (38.1%), married (57.1%), and who lived with their families (71.4%) (Table). They lived in the city of Sao Paulo (66.7%), had piped water, sewage and garbage collection in their homes (42.8%), were owners of their own houses (81.0%), which were all made of masonry material (Table). Seventeen patients (81.0%) had been using public health services. Of the workers (85.7%), 23.8% worked in cottages. Seventeen (81.0%) reported their income was enough for living. Eleven patients (52.4%) were working when the accident occurred. The more often affected anatomical regions were feet (42.9% of the patients) and hands (38.0%). Most patients (66.7%) carried out procedures at the site of the bite before searching for assistance, making use of a tourniquet, by applying alcohol, garlic, tobacco, and even performing incisions or suctions (or both).

**Table:** Data of the victims of snakebite, 2010-2012.

Variable	n	%
Sex		
Male	20	95.2
Female	1	4.8
Age (years)		
15-20	1	4.8
21-30	5	23.8
31-40	3	14.3
41-50	4	19.1
51-60	5	23.8
61-70	3	14.3
Schooling (concluded years of study)		
0	2	9.5
1-5	7	33.3
6-10	8	38.1
11 and more	4	19.1
Marital status		
Married	12	57.1
Single	8	38.1
Divorced	1	4.8
Who they lived with		
Alone	6	28.6
Family	15	71.4
Origin		
City of Sao Paulo	7	33.3
Other city of the State of Sao Paulo	14	66.7
Ownership of property		
Owner	17	81.0
Borrowed	3	14.2
Rented	1	4.8
Kind of property		
Masonry	21	100
Housing benefits: piped water and sewage and garbage collection		
All	9	42.8

The time between the accident and the arrival to the hospital was more than three hours for 11 (52.4%) patients. One of the patients has spent 34h in a health center before having properly treated in the specialized hospital.

The qualitative analysis of the interviews enabled us to expand the understanding of situations in which accidents occurred, as well as the understanding of the patients’ vulnerabilities. The lack of knowledge on the animal’s characteristics leads to accidents. In addition to this, patients recognized their self-carelessness and their own “fault” on the accident occurrence.

In relation to the animal identification, some patients reported that it is possible to identify venomous animals by the size of its head. However, other patients said that they were unaware of the difference between venomous and non venomous snakes.

Regarding assistance, three patients went immediately to the specialized hospital and 14 went previously to other health units closer to their homes. Four patients resorted to two health services before going to the specialized hospital. One of them said having waited for approximately 24h until being transferred to intensive health care in the same hospital. Some patients reported the lack of knowledge of the professional who attended them, and the fact that the doctor unconsidered the possibility of snakebite, only prescribing an anti-allergy drug. The nurses and technicians who took part of the staff were unfamiliar with the management of this kind of health problem, with exception of the specialized team of the hospital.

Regarding the actions taken after the accident, patients reported having cut, squeezed, and washed the site of the bite with alcohol or herbs, also applying tobacco and making use of a tourniquet, keeping the member bitten elevated. Only one of the patients did not carry out any further intervention on the bite site.

In relation to prevention, patients believed the use of boots, stockings, pants, leather blouses, gloves, and caps could have avoided the accident. One of the patients mentioned that, in general, people do not wear personal protective equipments. However, others declared using personal protective equipment, but because of the high temperature they often failed to do so. Regarding the perceptions on poisoning and serumtherapy, patients believed the venom could both kill and save. Thus, the animal must be preserved alive in the nature.

## DISCUSSION

The study showed elements that evidenced individual, social, and programmatic vulnerabilities: poor knowledge on snakes and on how to act preventively in relation to accidents, the non-use of personal protective equipments, the difficult access to health services, and, apparently, unprepared professionals to adequately assist such cases.

Although the sample of victims of bothropic accidents admitted in specialized hospitals is not considered representative, the results agree with the national literature.^5^ ^,^ ^18^ ^,^ ^19^ ^,^ ^21^ ^,^ ^23^ In this region, 113 victims of bothropic accidents were hospitalized in the same reported time.

Previous studies also observed the most affected anatomical regions in these accidents were hands and feet,^5^ ^,^ ^23^ and that most people carried out procedures at the site of the bite before searching for assistance,^18^ ^,^ ^19^ ^,^ ^22^ which shows a lack of adequate information, being considered a situation of individual vulnerability.

Most patients had a delay in the access of specialized intervention. The time is one critical element in the clinical evolution of accidents, being the most important factor in the prognosis.^14^ ^,^ ^21^ The delay observed in this study suggests programmatic vulnerability. Patients treated in less than three hours after the accident in general have a better clinical evolution.

The year of 1998 was of major advances, with the creation of the Brazilian Unified Health System (SUS) and the National Program for Control Accidents by Poisonous Animals (NPCAPA). These two classified accidents caused by poisonous animals as a compulsory notification with universal access to totally free treatment, and its respective guidelines, throughout the country as for HIV/Aids and tuberculosis, among other diseases. Both initiatives (SUS and NPCAPA) allowed the decentralization of the distribution of serumtherapy and the continuous training of health teams, which significantly reduced how many patients presented complications, sequelae, and deaths by snakebites across the country. However, because of the persistence of these vulnerabilities, we need to address its causes to suit future outcomes, since the amount of accidents and deaths caused by snakebites remains stable (around 28,000 and 100 per year, respectively) for more than a decade in Brazil.^4^

The bothropic venom has three main actions in the human accident: local acute inflammatory, coagulant, and haemorrhagic.^13^ ^,^ ^25^ In relation to the local clinical picture, we can associate edema with ecchymosis and pain. Few hours after the accident, bubbles on the bite site can arise. The main local complications are secondary infections, necrosis, compartmental syndrome, functional deficit, and amputation. The intensity and the extent of necrosis are strongly related to the use of tourniquet and to the delay on the beginning of the antivenom treatment. Soon after the accident, we need to maintain the patient at rest, clean the bite site, do not use topical substances, and carry the patient to a treatment center for antivenom application.^7^ ^,^ ^13^ ^,^ ^14^ ^,^ ^31^ We can classify bothropic accidents as mild, moderate, and severe. The dosage of antibotropic serum may increase with the severity of the accident (2-4 vials in mild, 4-8 in moderate, and 12 in severe cases).^14^

The analysis of sociodemographic characteristics and the occurrence of accidents showed the following elements of individual and social vulnerability: precarious schooling, low professional qualification, informal job, lack of access to adequate sanitation, and inadequate procedures after the accident. They may not only be interpreted as inherent attributes to individuals, but they are also linked to the place they occupy in society, determining specific conditions of access to a qualified life. The delay to transfer patients to health services with antivenom highlights the programmatic vulnerability. The limited number of people in this study only allows the inference that such elements may be constitutives of vulnerability.

In relation to the recognition of the animal, patients stated the size of the head is linked to the venomous animal. The appropriate way to identify the Brazilian venomous snakes is the presence of the loreal pit (which features the venomous serpent) and the shape of the tail: the presence of rattle determines *Crotalus* and the tail that gradually tapers determines the *Bothrops* genus.

In relation to health services, we found that most patients went to other health unit before going to a hospital specialized in accidents caused by venomous animals. Even thought more than 3,200 Brazilian municipalities have appropriately trained professionals and serumtherapy provision, the correct diagnosis, the appropriate health care, and the availability of specific serum must improve.

Some patients reported the lack of knowledge of health professionals and that the doctor disconsidered the possibility of snakebite, having prescribed an inadequate treatment. Health professionals should be trained to know the clinical effects of poisons to manage serumtherapy in a correct quantity, based on the evaluation of severity by the time of admission in the health service, being one of the aspects in which nurses must acquire competence. In this study, deficiencies in the access to health services, the lack of serumtherapy in health services, in addition to limitations on the technical background of health professionals denote elements of programmatic vulnerability.

Regarding the actions taken after accidents, patients reported a series of incorrect procedures showing individual and social vulnerability, and the deficiency of the NPCAPA, which also denotes programmatic vulnerability, suggested by the lack of antivenom and properly trained teams in some public health services. The Brazilian Ministry of Health make the following recommendations: to wash the site of the bite, preferably with soap and water, keep the victims at rest, and immediately transfer them to a health service with serumtherapy, excluding the use of tourniquets or methods such as drilling, cutting, burning, squeezing, sucking, neither putting herbs, coffee powder nor earth.^12^ ^,^ ^14^ ^,^ ^16^

Serumtherapy is the only specific treatment and, when indicated it should be administered in health services under medical supervision. Intravenous administrations are preferred because they provide greater speed of distribution and bioavailability of antivenom in the human body. Health care professionals need to know the clinical effects of poisons to indicate the right antivenom dosage, based on the evaluation of severity, a situation in which nurses can certainly support.^13^ ^,^ ^14^

On perceptions concerning prevention actions, patients knew the need to wear appropriate clothing when exposed to places with wood, for example. According to the Brazilian Ministry of Health, the use of protective clothing such as leather gloves, closed shoes, boots, and ankle boots, should be mandatory in rural activities.^12^ ^,^ ^16^ ^,^ ^31^ Using boots or leggings would avoid more than two thirds of the number of accidents.^13^ ^,^ ^31^ However, in general, people do not use personal protective equipments, an element of individual and social vulnerability. In Brazil, all companies need to provide appropriate protective equipment free of charge and in perfect working conditions to their employees.^31^

We should not understand vulnerable behaviors by people’s voluntary action since they comprise the social position of individuals, their living and working conditions, cultural aspects, degree of consciousness, and empowerment to transform them.^17^ Which means understanding behaviors and attitudes and paying attention in the political economic structural aspects, enabling more comprehensive interventions to meet people’s health needs.^27^

In synthesis, all patients’ reports showed elements that denote individual and social vulnerabilities: poor knowledge on snakes and on how to act preventively in relation to accidents, and the non-use of personal protective equipments. In the programmatic dimension, we noted the difficulty of access to the health services and unprepared professionals.

The present study has some limitations. The results are based on the perception of a sample of patients admitted on the hospital specialized in accidents caused by venomous animals, which limits the findings to a particular location. As snakebites are a globally neglected health problem, this study considers the patients’ point of views to expand the knowledge on the subject. We should account for some bias, in the sense of undesirable skews. Therefore, we cannot generalize these results. Despite such limitations, the sample allowed us to reach systematic repetition of statements when responses were similar among participants. This article is the first to analyze snakebites based on the concept of vulnerability.

Elements of vulnerability in individual, social, and programmatic dimensions suggest the need to improve programs for snakebites control. The access to health services should be a government commitment settled in the constitution of the Brazilian Health System, since health is a right of all citizens. Our approach is innovative and associated with the attempt to understand the patients’ needs to achieve the best in health care services by going beyond the normative character and incorporating elements related to sociocultural aspects.

## Footnotes

a Ministério da Saúde, Sistemas de Informação de Agravos de Notificação - SINAN. Acidentes por animais peçonhentos. Brasília (DF): Ministério da Saúde; 2004 [cited 2015 Feb 12]. Available from: http://dtr2004.saude.gov.br/sinanweb/tabnet/tabnet?sinannet/animaisp/bases/animaisbrnet.def
